# A Rare Case of Collecting Duct Carcinoma: A Comprehensive Analysis Using Ultrasound, Computed Tomography, and Histopathological Examination

**DOI:** 10.7759/cureus.67463

**Published:** 2024-08-22

**Authors:** Sanjaykanth B, Jasvant Ram Ananthasayanam, Ajina Sam, Karpagam R K, Karthik Krishna Ramakrishnan

**Affiliations:** 1 Radiodiagnosis, Saveetha Medical College and Hospitals, Saveetha Institute of Medical and Technical Sciences, Saveetha University, Chennai, IND

**Keywords:** magnetic resonance imaging, renal cell carcinoma (rcc), computed tomography, non-clear renal cell carcinoma, collecting duct renal cell carcinoma

## Abstract

Collecting duct renal cell carcinoma (cdRCC) is an exceptionally rare and aggressive subtype of renal cell carcinoma, accounting for approximately 1% of all renal tumors. This case is notable due to the comprehensive use of multi-modality imaging and detailed histopathological examination, offering valuable insights into the diagnostic challenges and management of this rare condition. A 64-year-old male presented with progressive right flank pain, hematuria, and decreased urine output. Imaging studies revealed a hypoechoic lesion in the right kidney, predominantly located in the hilar and perihilar regions, suggestive of a malignant renal tumor. Further diagnostic evaluation, including a right radical nephrectomy, was performed. The histopathological examination of the resected tissue confirmed the diagnosis of cdRCC, characterized by a tubulopapillary growth pattern, significant pleomorphism, and sarcomatoid changes. Immunohistochemical analysis showed strong positivity for epithelial membrane antigen and CK7, confirming the aggressive nature of the tumor. This case underscores the importance of early diagnosis and a comprehensive diagnostic approach to managing cdRCC. Despite advances in imaging techniques, a definitive diagnosis often relies on histopathological and immunohistochemical analysis. The aggressive nature of cdRCC and its generally poor prognosis highlight the need for prompt and accurate diagnosis to potentially improve patient outcomes. This report adds to the limited literature on cdRCC, emphasizing the challenges and considerations in diagnosing and managing this rare form of renal carcinoma.

## Introduction

Collecting duct renal cell carcinoma (cdRCC) is a highly aggressive and rare subtype of renal cell carcinoma. Traditionally believed to arise from the collecting ducts of Bellini within the renal medulla, cdRCC is characterized by its late-stage presentation and poor prognosis. Nearly half of the patients are diagnosed at advanced stages, indicating significant disease progression at the time of detection [1]. This carcinoma typically originates in the distal collecting duct and shares histopathological features with urothelial carcinoma, complicating differential diagnosis. The aggressive nature of cdRCC is underscored by its rapid progression and high metastatic potential. A substantial percentage of patients present with distant metastases at diagnosis, and the majority experience poor outcomes within a short period [2]. The lack of specific clinical and radiological markers further complicates early detection and accurate diagnosis. Common imaging findings, such as medullary location, weak and heterogeneous enhancement, and involvement of the renal sinus, are not unique to cdRCC and often overlap with other renal tumors. These characteristics, combined with the tumor's rarity, pose significant diagnostic challenges, necessitating a high index of suspicion and thorough evaluation.

Histologically, cdRCC often exhibits a tubulopapillary growth pattern with notable cellular pleomorphism and occasional sarcomatoid differentiation. Immunohistochemical profiling, which may reveal markers such as epithelial membrane antigen and CK7, aids in the differentiation from other renal and urothelial carcinomas. However, the overlap in these features necessitates comprehensive pathological and radiological analysis to reach a definitive diagnosis [3,4]. Given the aggressive course and poor prognosis associated with cdRCC, early and accurate diagnosis is critical. The management of this rare carcinoma often involves a combination of surgical and chemotherapeutic interventions, although the optimal treatment approach remains under investigation due to limited clinical data. This study aims to provide a comprehensive review of the clinical, pathological, immunohistochemical, and radiological aspects of cdRCC, emphasizing the challenges in diagnosis and implications for patient management [5,6].

## Case presentation

A 64-year-old male patient presented to the urology outpatient department with complaints of progressive right flank pain for the past 15 days. The pain was described as dull and aching, with occasional sharp exacerbations. The patient also reported hematuria and burning micturition for the last 10 days, along with decreased urine output and three episodes of loose stools. He denied any symptoms of melena, fever, vomiting, or chills.

On physical examination, the patient appeared moderately built and nourished. Vital signs were stable. An abdominal examination revealed mild tenderness in the right flank region without any palpable mass. There were no signs of jaundice, peripheral edema, or lymphadenopathy. Significant findings included the presence of hematuria and symptoms suggestive of urinary tract involvement.

**Figure 1:**
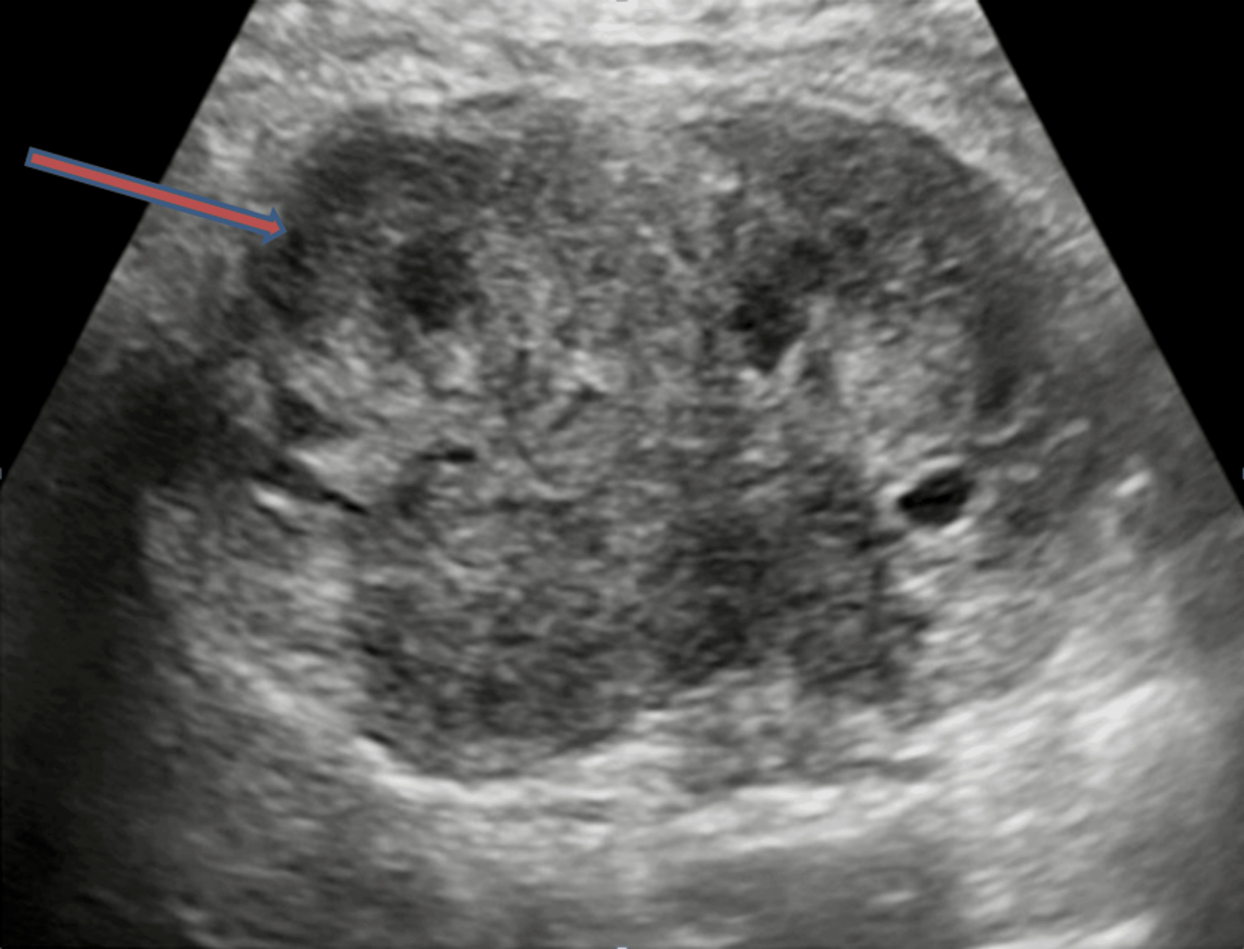
The ultrasound image demonstrates a heterogeneous hypoechoic lesion predominantly located in the hilar and perihilar regions of the kidney. The mixed echogenicity within the lesion indicates varying tissue densities. The hypoechoic nature suggests less dense tissue than the surrounding renal parenchyma. The central location potentially affects the renal pelvis and major blood vessels, indicative of a malignant tumor.

**Figure 2:**
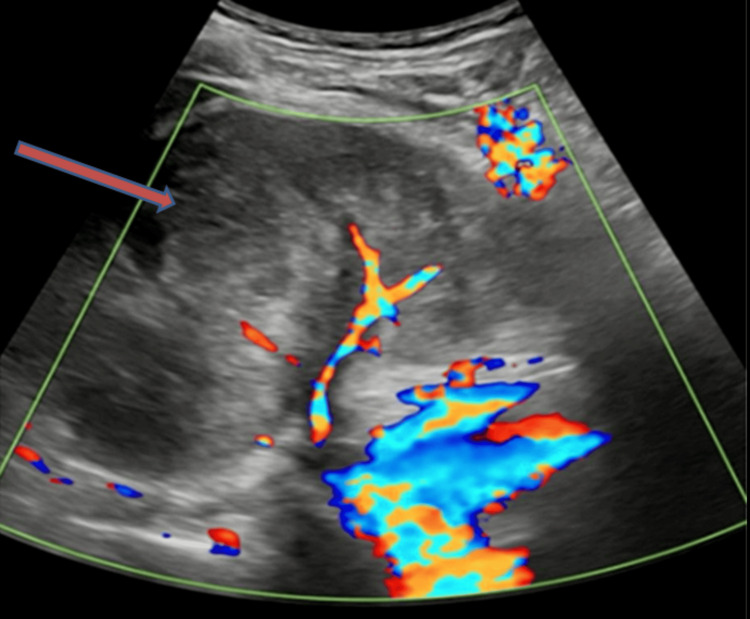
The color Doppler ultrasound image reveals a lack of significant color uptake within the lesion, while the surrounding renal parenchyma shows normal blood flow. The absence of color uptake suggests the lesion is avascular or has very low blood flow, characteristic of collecting duct carcinoma. This lack of vascularity can indicate necrosis or poor perfusion, associated with aggressive malignancies.

**Figure 3:**
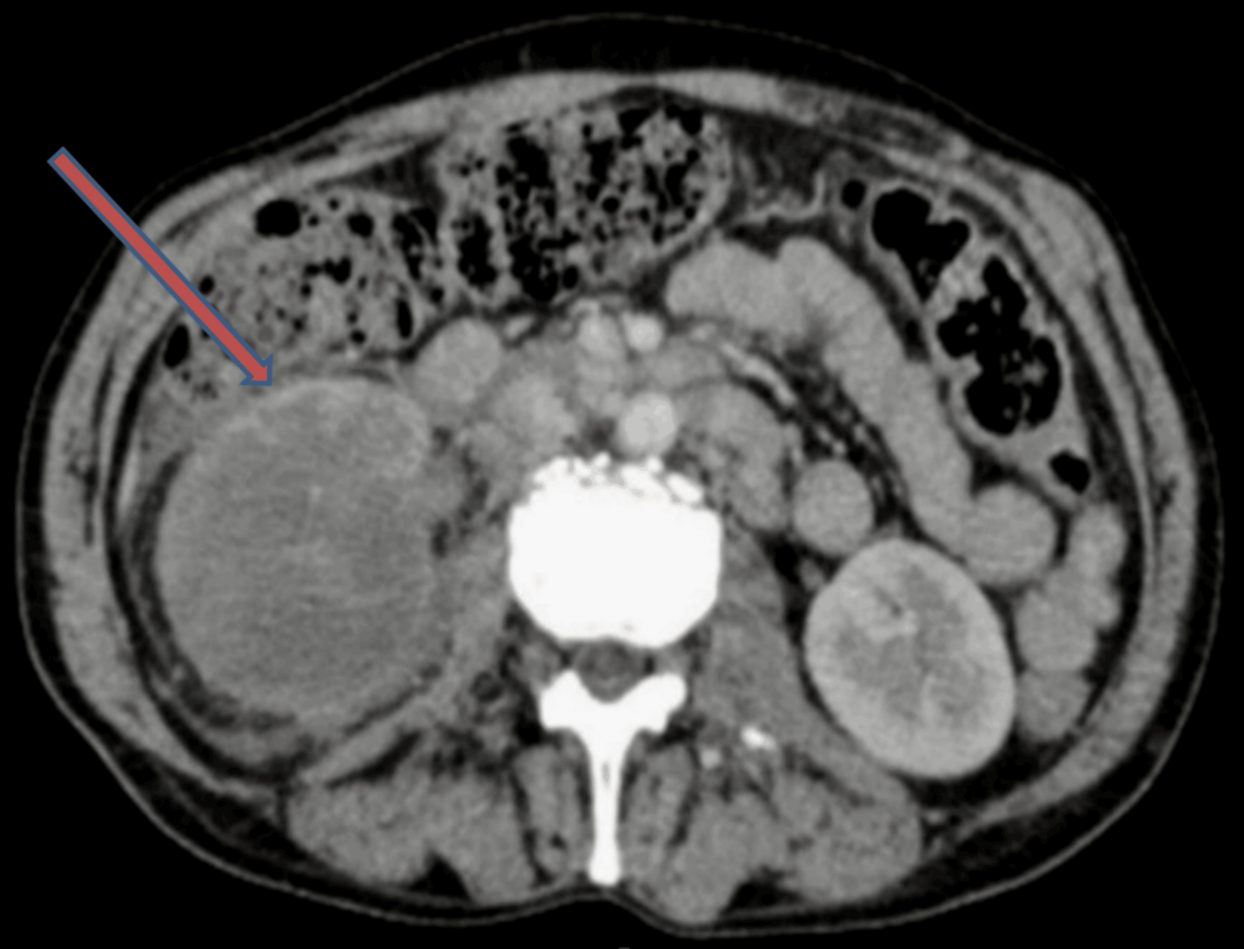
The axial contrast-enhanced CT image shows a peripherally enhancing mass with predominant hypoenhancement in the hilar and perihilar regions of the right kidney. This pattern of peripheral enhancement with central hypoenhancement is indicative of a malignant lesion, as it suggests the presence of a viable tumor rim with central necrosis or poor perfusion, characteristic of collecting duct carcinoma.

**Figure 4:**
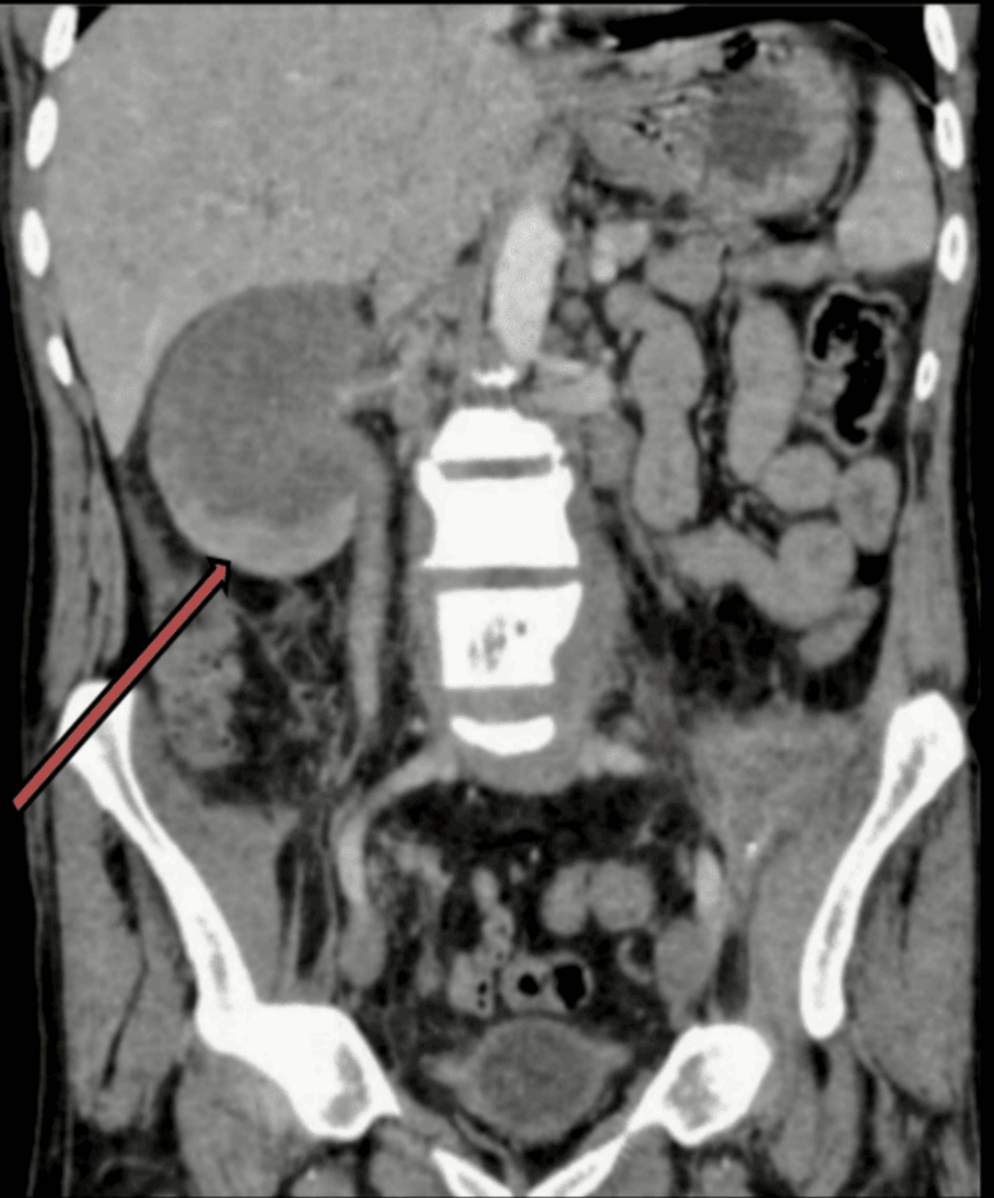
The coronal post-contrast CT image highlights the extent of the mass in the right kidney, emphasizing its peripheral enhancement. This view demonstrates the replacement of normal renal parenchyma in the hilar and perihilar regions by the tumor. The peripheral enhancement and central hypoenhancement patterns further support the suspicion of a malignant process, such as collecting duct carcinoma.

The initial diagnostic evaluation included abdominal ultrasonography, which demonstrated a heterogeneously hypoechoic lesion in the hilar and perihilar regions of the right kidney, measuring approximately 6.2 × 5.2 cm. This was suggestive of a malignant renal mass (Figures 1, 2). A contrast-enhanced computed tomography (CT) scan of the abdomen and pelvis further characterized the mass as a peripherally enhancing, centrally hypoenhancing lesion in the hilar and perihilar right kidney. The imaging findings raised suspicion for cdRCC (Figures 3, 4).

**Figure 5:**
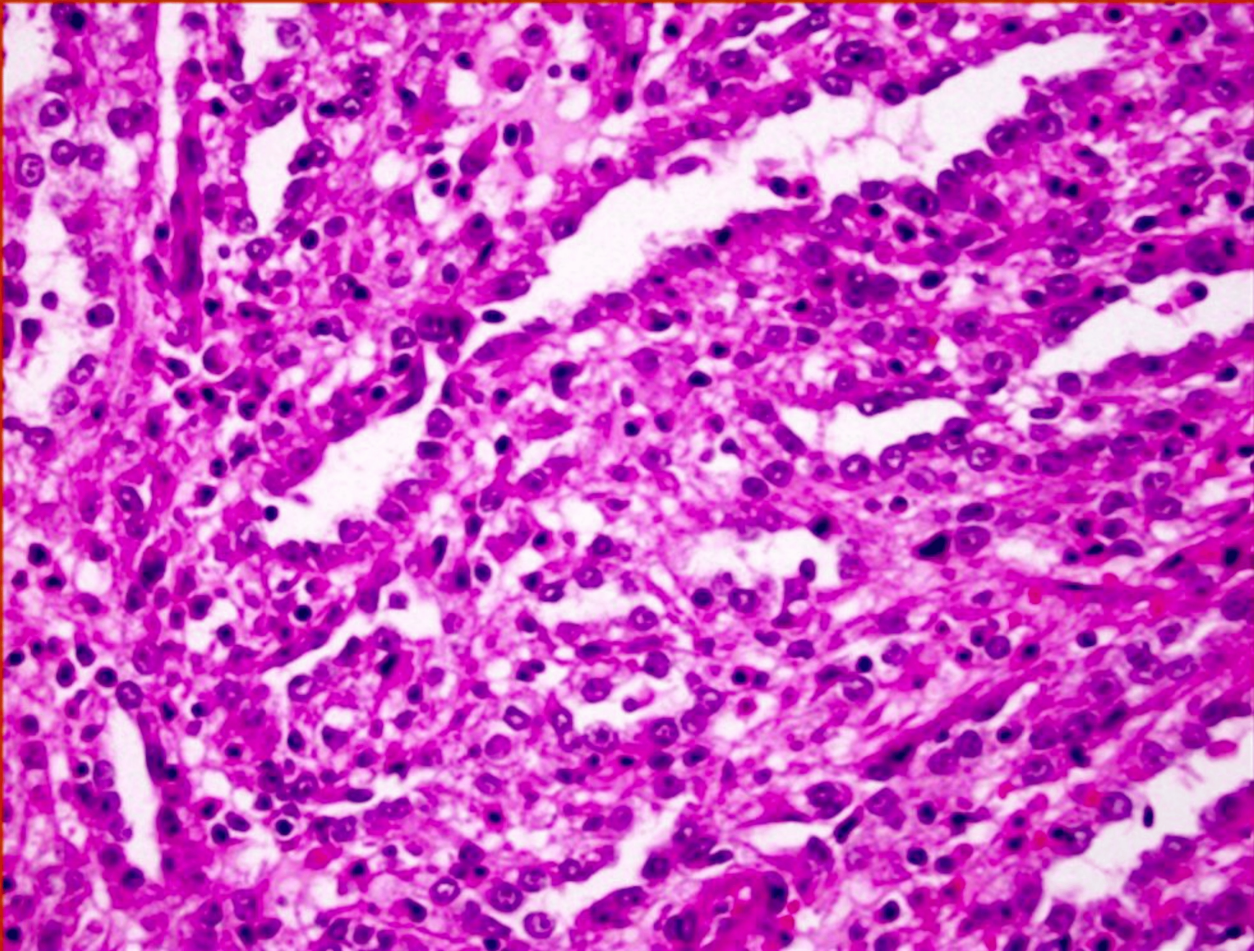
The tissue biopsy sent for histopathological examination revealed a tubulopapillary growth pattern with significant pleomorphism of the neoplastic cells and local sarcomatoid changes. These features are visible in the histopathological image, which shows the disorganized architecture and marked variation in cell shape and size. Based on these characteristics, the tumor was classified as nuclear grade IV according to the WHO/ISUP classification. Immunohistochemical analysis demonstrated that the tumor cells were strongly positive for epithelial membrane antigen and cytokeratin (CK) 7, confirming the diagnosis of collecting duct carcinoma. WHO/ISUP: World Health Organization/International Society of Urological Pathology

Due to the rarity of cdRCC and its overlapping features with other renal tumors, there were significant challenges in arriving at a definitive diagnosis preoperatively. The lack of specific radiologic features unique to cdRCC complicated the diagnostic process. The primary diagnosis of cdRCC was established postoperatively through histopathological examination, which revealed a tubulopapillary growth pattern with significant pleomorphism of neoplastic cells and local sarcomatoid changes, as shown in Figure 5. Immunohistochemical analysis showed strong positivity for epithelial membrane antigen and CK7, confirming the diagnosis. Other renal tumors, such as urothelial carcinoma, were considered and excluded based on histopathological and immunohistochemical findings.

**Figure 6:**
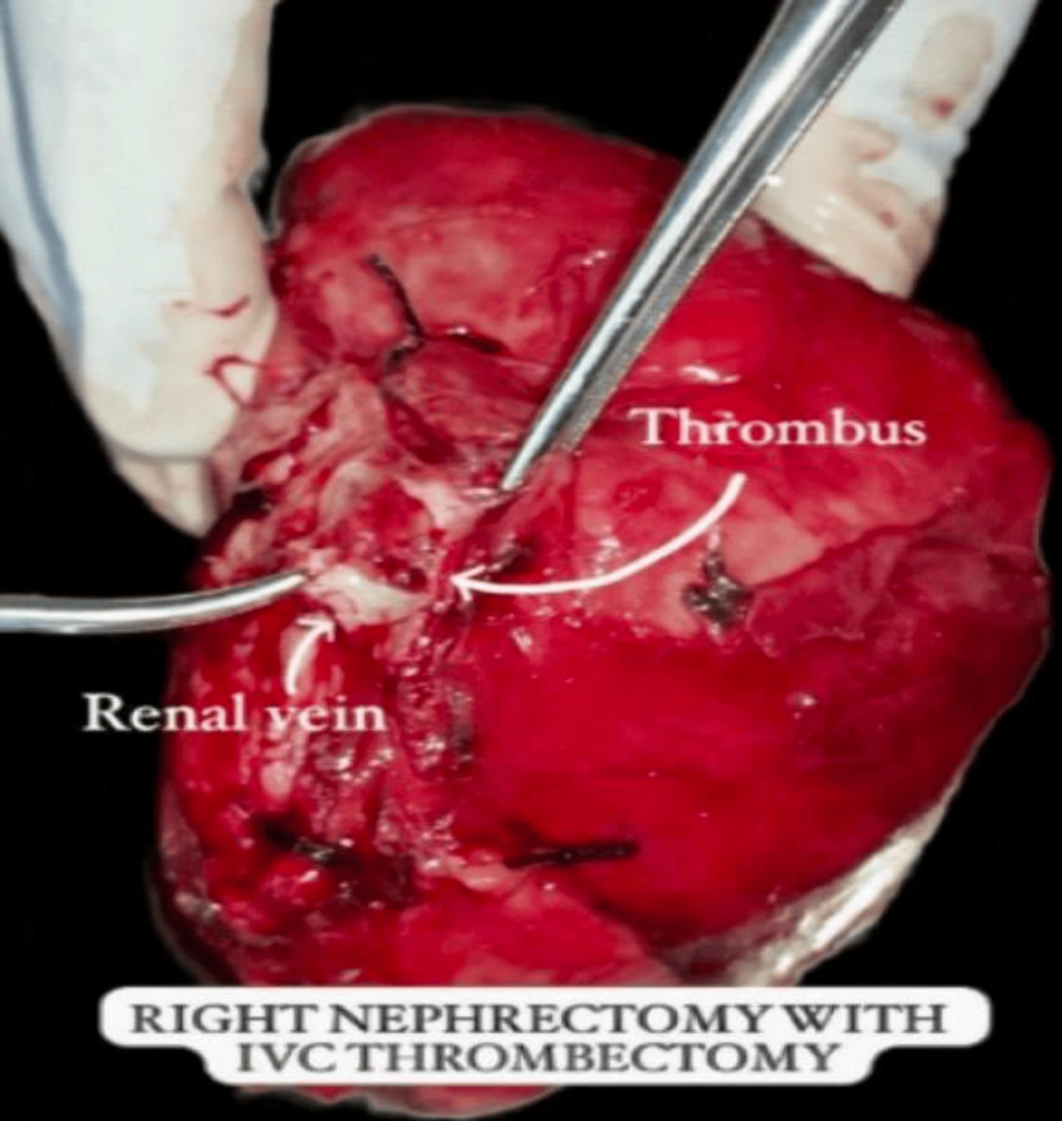
The intraoperative image shows the right nephrectomy specimen with an associated thrombus being removed from the renal vein. This image highlights the surgical procedure of right radical nephrectomy combined with thrombectomy, addressing both the primary tumor and the associated thrombus.

**Figure 7:**
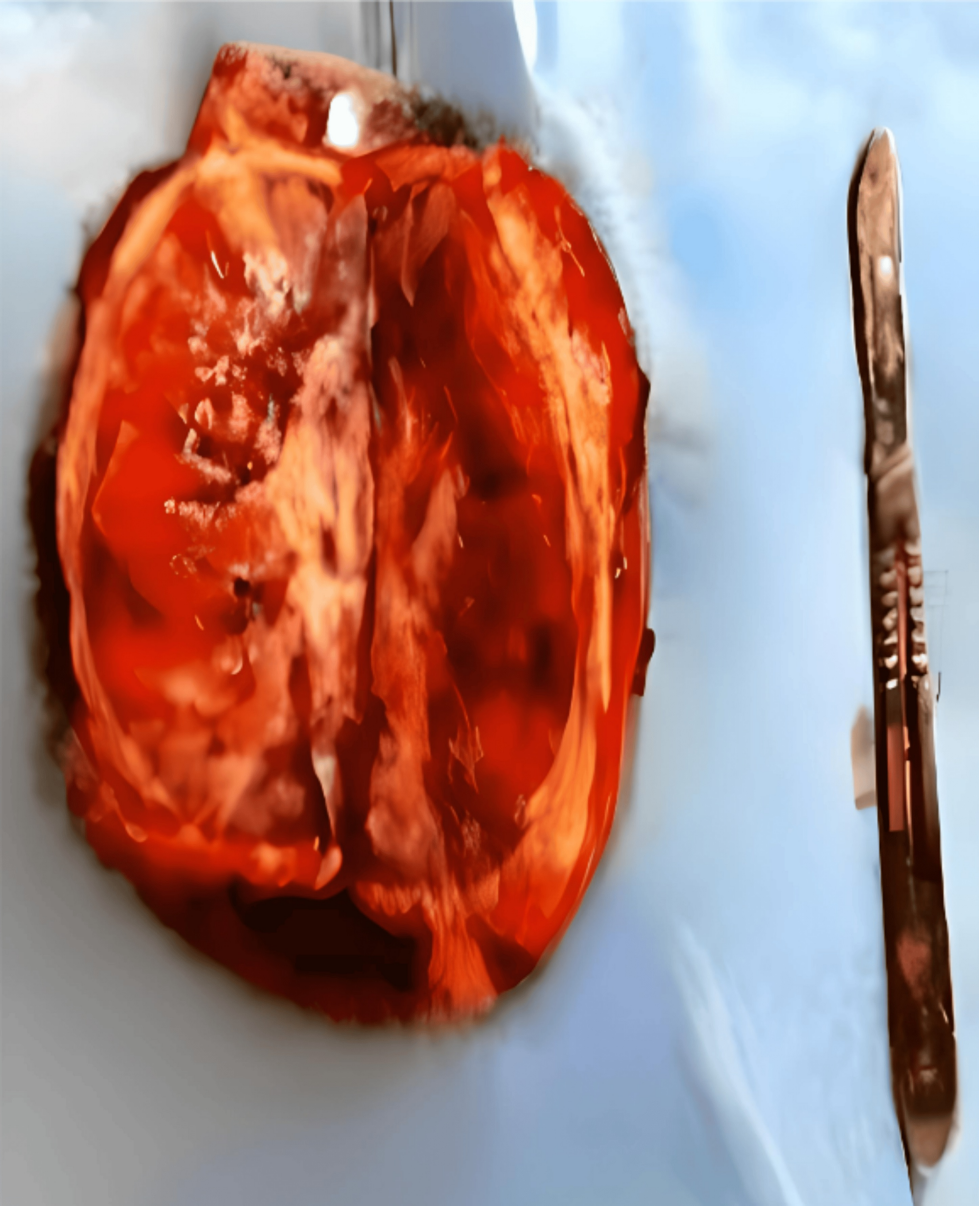
The specimen image displays the resected kidney following a right radical nephrectomy. The macroscopic examination reveals a tan-yellow, well-circumscribed, soft tumor occupying almost the entire upper pole of the kidney. This appearance is consistent with the typical presentation of a tumor in collecting duct carcinoma, indicating an extensive malignancy that necessitated surgical intervention.

The patient's prognosis was poor due to the aggressive nature of cdRCC, with a high potential for metastatic spread. The tumor was classified as nuclear grade IV according to the WHO/ISUP (World Health Organization/International Society of Urological Pathology) classification. The patient underwent a right radical nephrectomy with thrombectomy due to the presence of an associated thrombus in the renal vein. The nephrectomy specimen revealed a tan-yellow, well-circumscribed tumor occupying almost the entire upper pole of the kidney (Figures 6, 7).

The primary surgical intervention involved the complete removal of the kidney along with the tumor. Post-surgery, the patient had a good recovery, with no immediate complications reported. Follow-up assessments, including imaging studies and laboratory tests, showed no signs of disease recurrence or progression. The patient's renal function remained stable, and there were no adverse effects from the surgery. The patient adhered well to the postoperative care plan, and the tolerability of the intervention was satisfactory. The follow-up protocol included regular imaging studies, such as ultrasound and CT scans, every three months during the first year post-surgery, followed by biannual assessments. These imaging studies confirmed the absence of new lesions or metastases, indicating that the patient remained in remission. Additionally, laboratory tests, including renal function tests and tumor markers, were monitored periodically, all of which remained within normal limits.

The comprehensive case presentation highlights the diagnostic and therapeutic challenges associated with managing cdRCC, a rare and aggressive renal carcinoma. The importance of a thorough diagnostic approach, including advanced imaging and histopathological confirmation, is emphasized to improve patient outcomes. The detailed follow-up and continuous monitoring were crucial in ensuring early detection of any potential recurrence, thereby allowing timely intervention. This approach has contributed significantly to the patient's favorable prognosis and overall recovery, underscoring the necessity of meticulous postoperative care and monitoring in managing cdRCC. Further follow-up and detailed documentation of patient progress and outcomes remain essential for understanding the long-term prognosis and effectively managing potential complications.

## Discussion

The presented case report of cdRCC underscores several strengths in its diagnostic and therapeutic approach. The use of advanced imaging modalities, such as contrast-enhanced CT and ultrasonography, played a pivotal role in identifying the characteristics of the renal mass. These imaging techniques allowed for the visualization of the lesion's heterogeneity and the assessment of its extent, which are crucial for staging and treatment planning. Additionally, the histopathological examination, supported by immunohistochemical analysis, confirmed the diagnosis of cdRCC, highlighting the tumor's tubulopapillary growth pattern and the presence of significant pleomorphism and sarcomatoid changes. This comprehensive diagnostic approach is a strength, as it facilitates an accurate diagnosis, which is essential for guiding treatment [5].

However, the case also highlights several limitations, particularly related to the rarity of cdRCC. The lack of specific radiological features unique to cdRCC complicates its differentiation from other renal tumors, such as urothelial carcinoma and other non-clear cell renal cell carcinoma (ncRCC) subtypes. This diagnostic ambiguity often necessitates invasive procedures such as biopsies for a definitive diagnosis, which may not always be feasible or safe. Moreover, the rarity of cdRCC poses challenges in establishing standardized treatment protocols, as there is limited clinical data and few large-scale studies available [6]. This rarity also complicates access to newer therapeutic options and clinical trials, limiting the treatment landscape primarily to conventional methods such as nephrectomy and platinum-based chemotherapy, which may not be optimally effective​​.

In the broader medical literature, cdRCC is recognized as a highly aggressive and rare subtype of renal cell carcinoma, accounting for less than 2% of all renal tumors. The disease is often diagnosed at an advanced stage, with a significant proportion of patients presenting with metastatic disease. This advanced stage at diagnosis contributes to the poor prognosis associated with cdRCC, with a median overall survival of less than 12 months for metastatic cases. The disease's aggressive nature is further underscored by frequent distant metastases and a high cancer-specific mortality rate compared to other renal cell carcinoma subtypes. Conventional treatments, such as surgery and platinum-based chemotherapy, have shown limited efficacy, particularly in advanced stages. Recent clinical trials have explored targeted therapies, such as cabozantinib, and immunotherapies, such as avelumab, with mixed results. While these newer treatments have shown some promise, their effectiveness remains limited, and the prognosis for cdRCC patients continues to be poor [7]​.

The scientific rationale for the observed clinical features and treatment challenges in cdRCC lies in its molecular and genetic characteristics. Genomic studies have revealed mutations in genes such as *SETD2*, *CDKN2A*, *SMARCB1*, and *NF2*, which may contribute to the tumor's aggressive phenotype. Additionally, the high prevalence of immune cells within the tumor microenvironment suggests potential avenues for immunotherapy. However, the rarity of cdRCC complicates the conduct of large-scale studies, which are necessary to establish the efficacy of new treatments. The current understanding of cdRCC's pathogenesis and treatment is still evolving, with a need for further research to uncover novel therapeutic targets and improve patient outcomes.

The primary lesson from this case report is the critical importance of early and accurate diagnosis in managing rare and aggressive tumors such as cdRCC [8]. The case highlights the need for a multidisciplinary approach that includes advanced imaging, detailed histopathological analysis, and consideration of emerging therapies, such as immunotherapy, to potentially improve outcomes. Despite the challenges posed by the rarity and aggressive nature of cdRCC, comprehensive diagnostic evaluations and a willingness to explore new treatment modalities are essential for optimizing patient care. Further research and multi-institutional collaborations are necessary to advance the understanding and treatment of this rare renal carcinoma.

## Conclusions

This case report highlights the diagnostic challenges and aggressive nature of collecting duct carcinoma (CDC), a rare subtype of renal cell carcinoma. Despite advances in imaging techniques such as ultrasound and contrast-enhanced CT, CDC remains difficult to distinguish from other renal tumors preoperatively. Histopathological and immunohistochemical analyses are crucial for a definitive diagnosis. The presented case underscores the importance of early detection and comprehensive diagnostic evaluation to improve prognosis. Surgical intervention remains a key component of management, although the prognosis for CDC is generally poor due to its aggressive behavior and high metastatic potential at the time of diagnosis. Further research and clinical awareness are essential to better understand and manage this rare and lethal form of renal carcinoma.

## References

[1] Ciszewski S, Jakimów A, Smolska-Ciszewska B (2015). Collecting (Bellini) duct carcinoma: a clinical study of a rare tumour and review of the literature. Can Urol Assoc J.

[2] Mansoor M, Young-Speirs M, Ren B (2022). Extrarenal renal cell carcinoma arising in the kidney proximity but without an identifiable renal primary - an intriguing dilemma: report of three cases and review of the literature. Histopathology.

[3] Tang C, Zhou Y, Ge S, Yi X, Lv H, Zhou W (2021). Incidence, clinical characteristics, and survival of collecting duct carcinoma of the kidney: a population-based study. Front Oncol.

[4] Milowsky MI, Rosmarin A, Tickoo SK, Papanicolaou N, Nanus DM (2002). Active chemotherapy for collecting duct carcinoma of the kidney: a case report and review of the literature. Cancer.

[5] Wright JL, Risk MC, Hotaling J, Lin DW (2009). Effect of collecting duct histology on renal cell cancer outcome. J Urol.

[6] Sui W, Matulay JT, Robins DJ (2017). Collecting duct carcinoma of the kidney: disease characteristics and treatment outcomes from the National Cancer Database. Urol Oncol.

[7] Pickhardt PJ, Siegel CL, McLarney JK (2001). Collecting duct carcinoma of the kidney: are imaging findings suggestive of the diagnosis?. AJR Am J Roentgenol.

[8] Kato H, Kanematsu M, Yokoi S, Miwa K, Horie K, Deguchi T, Hirose Y (2011). Renal cell carcinoma associated with Xp11.2 translocation/TFE3 gene fusion: radiological findings mimicking papillary subtype. J Magn Reson Imaging.

